# Filamins but Not Janus Kinases Are Substrates of the ASB2α Cullin-Ring E3 Ubiquitin Ligase in Hematopoietic Cells

**DOI:** 10.1371/journal.pone.0043798

**Published:** 2012-08-20

**Authors:** Isabelle Lamsoul, Monique Erard, Peter F. M. van der Ven, Pierre G. Lutz

**Affiliations:** 1 CNRS, IPBS (Institut de Pharmacologie et de Biologie Structurale), 205 route de Narbonne BP64182, F-31077 Toulouse, France; 2 Université de Toulouse, UPS, IPBS, F-31077 Toulouse, France; 3 Department of Molecular Cell Biology, Institute of Cell Biology, University of Bonn, Bonn, Germany; Emory University, United States of America

## Abstract

The ASB2α protein is the specificity subunit of an E3 ubiquitin ligase complex involved in hematopoietic differentiation and is proposed to exert its effects by regulating the turnover of specific proteins. Three ASB2α substrates have been described so far: the actin-binding protein filamins, the Mixed Lineage Leukemia protein, and the Janus kinases 2 and 3. To determine the degradation of which substrate drives ASB2α biological effects is crucial for the understanding of ASB2α functions in hematopoiesis. Here, we show that neither endogenous nor exogenously expressed ASB2α induces degradation of JAK proteins in hematopoietic cells. Furthermore, we performed molecular modeling to generate the first structural model of an E3 ubiquitin ligase complex of an ASB protein bound to one of its substrates.

## Introduction

Ubiquitin-mediated protein degradation is the main mechanism for controlled proteolysis in Eukaryotes and ensures that specific protein functions are turned off at the right time and in the right place through the selective targeting of proteins to proteasome. Ankyrin repeat-containing protein with a suppressor of cytokine signaling box (SOCS) 2 (ASB2) proteins are the specificity subunits of multimeric E3 ubiquitin ligases of the cullin 5-RING ligase family [1], [2], [3], [4] suggesting that ASB2 proteins exert their effects by regulating the turnover of specific proteins. The *ASB2* gene encodes two different isoforms: a hematopoietic-type involved in hematopoietic differentiation, ASB2α [5], [6], and a muscle-type involved in myogenic differentiation, ASB2β [3]. Although ASB2α transcripts were initially identified in acute promyelocytic leukemia (APL) cells induced to differentiate by retinoic acid, they are expressed in normal hematopoietic cells [5], [7], [8] and so ASB2α is likely to be relevant during hematopoiesis. Importantly, *ASB2* is a transcriptional target of the oncoproteins, PML-RARα [5], [9], PLZF-RARα [10] and AML1-ETO (JHA Martens and HG Stunnenberg, personal communication), which act as transcriptional repressors suggesting that *ASB2* repression may participate in the leukemogenesis process. *ASB2* is not only activated upon retinoic acid-differentiation of PML-RARα-expressing APL cells but also upon granulocytic maturation of non-APL acute myeloid leukemia treated with retinoic acid in combination with the inhibitor of lysine-specific demethylase 1 tranylcypromine [11]. This together with the fact that ASB2α knockdown in myeloid leukemia cells delays retinoic acid-induced differentiation [6] indicate that *ASB2* is one of the genes associated with induced-differentiation of myeloid leukemia cells.

We have showed that ASB2α E3 ubiquitin ligase activity triggers polyubiquitylation of the actin-binding protein filamins (FLNa, FLNb and FLNc) leading to their proteasome-mediated degradation [6], [12], [13]. Furthermore, the Mixed Lineage Leukemia (MLL) protein, an activator of Hox genes and a regulator of hematopoietic stem cell self-renewal and hematopoietic progenitor proliferation, is another substrate of ASB2α in hematopoietic cells [14]. Importantly, MLL fusion proteins escape from ASB2α-mediated degradation; this likely contributes to leukemogenesis [14]. Moreover, the Janus kinases (JAK) 2 and 3 that are crucial for cytokine receptor signaling during hematopoiesis were proposed to be substrates of a non-canonical E3 ubiquitin ligase complex including ASB2α and the F-box containing protein Skp2 [15], [16]. Since these results were obtained following ectopic expression in epithelial cells, the validation of JAK proteins as direct substrates of ASB2α in hematopoietic cells remains to be established.

In this report, we show that ASB2α does not induce degradation of JAK proteins in hematopoietic cells and provide the structural model of the ASB2α E3 ubiquitin ligase complex bound to one of its substrates.

## Results and Discussion

**Figure 1:**
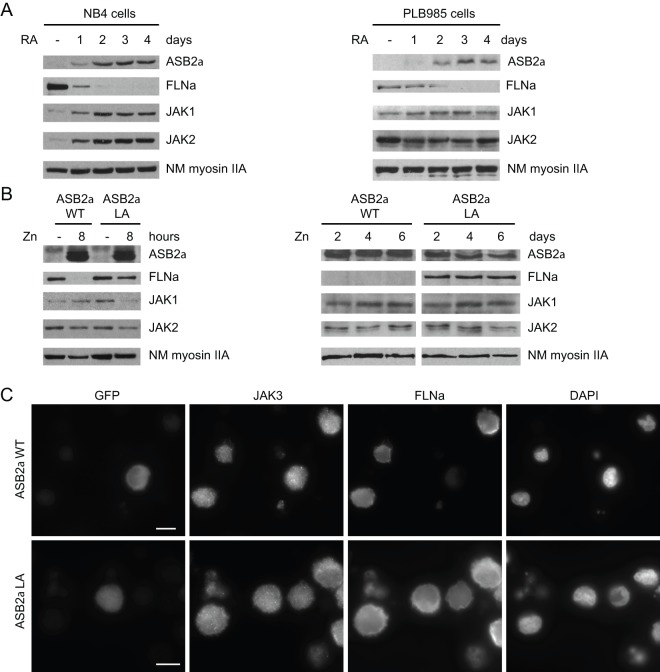
ASB2α induced degradation of FLNa but not of JAK1 and JAK2 in acute myeloid leukemia cells. (A) Expression of ASB2α, FLNa, JAK1, JAK2 and non-muscle myosin IIA heavy chain (NM myosin IIA) in differentiating leukemia cells. Promyelocytic NB4 and myeloblastic PLB985 cells were left untreated (-) or were treated with 10^−6^ M of *all-trans* retinoic acid (RA) as indicated. Differentiation was assessed by cell morphology on May-Grünwald-Giemsa-stained cytospins, and quantification of the percentage of cells with nitro blue tetrazolium deposits (not shown). (B) ASB2α E3 ubiquitin ligase activity triggers degradation of FLNa but not of JAK1 and JAK2 in myeloid leukemia cells. PLB985/MT-FlagASB2α (ASB2αWT) and PLB985/MT-FlagASB2αLA (ASB2αLA) cells were left untreated (-) or were treated for 8, 48, 96 and 144 hours with 75 μM or 85 μM ZnSO_4_, respectively to induce the expression of equivalent amounts of ASB2α proteins. In A and B, 10-μg aliquots of the protein extracts were separated by SDS-PAGE and immunoblotted for ASB2α, FLNa, JAK1, JAK2 and NM myosin IIA. (C) U937 cells were imaged 20 hours after nucleofection with GFP-ASB2α or GFP-ASB2αLA expression vector. Cells were fixed and stained for JAK3 and FLNa. Scale bar represents 10 μm.

To determine the degradation of which substrate drives ASB2α biological effects in hematopoiesis is crucial for the understanding of ASB2α functions. Because ASB2α was originally identified as induced by retinoic acid in acute myeloid leukemia cells [5], [7], we investigated the expression of JAK proteins known to be expressed in these cells [17]. Although endogenous ASB2α was induced upon retinoic acid-treatment of promyelocytic NB4 cells and myeloblastic PLB985 cells, no loss of JAK1 or JAK2 were observed (Figure 1A). In contrast, FLNa expression was drastically decreased and barely detected 48 and 72 hours after retinoic acid treatment of NB4 and PLB985 cells, respectively. These suggest that endogenous ASB2α did not regulate the turnover of JAK proteins during induced-differentiation of myeloid leukemia cells. We next investigated the expression of JAK1 and JAK2 in PLB985 cells induced to express wild-type ASB2α or the E3 ubiquitin ligase defective mutant ASB2αLA. Although wild-type ASB2α induced acute degradation of FLNa in 8 hours, levels of JAK1 and JAK2 proteins remained unchanged in cells expressing wild-type ASB2α (Figure 1B, left panels). Furthermore, sustained expression of wild-type ASB2α had no impact on the levels of the JAK1 and JAK2 (Figure 1B, right panels). Using U937 human myelomonocytic cells, we next investigated whether ASB2α induces degradation of JAK3. U937 cells were transiently transfected with GFP-ASB2α or GFP-ASB2αLA expression vectors and analyzed by immunofluorescence microscopy to visualize at the single-cell level the impact of ASB2α expression on JAK3 and FLNa. As shown in Figure 1C, ASB2α did not induce degradation of JAK3 while inducing degradation of FLNa. Altogether, these demonstrated that ASB2α does not induce degradation of JAK proteins in myeloid cells.

**Figure 2:**
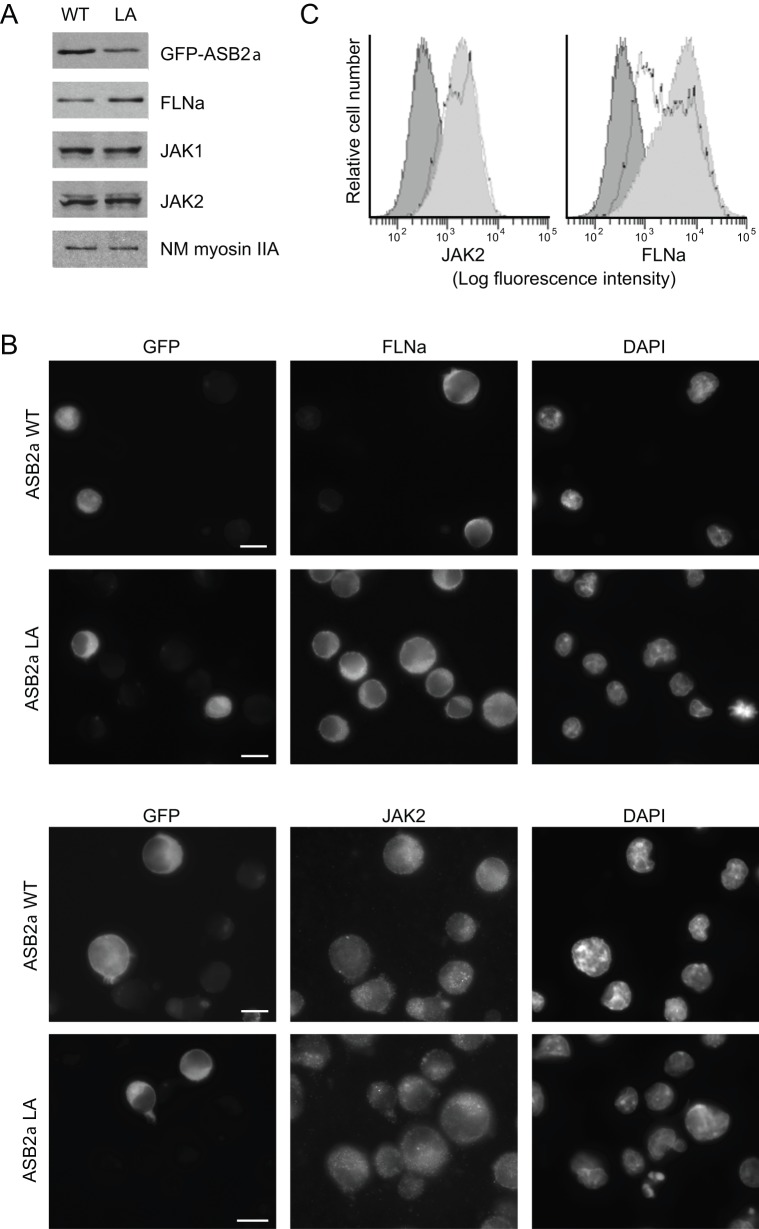
ASB2α induced degradation of FLNa but not of JAK1 and JAK2 in Ba/F3 murine pro-B cells. (A and B) Ba/F3 cells were nucleofected with the GFP-ASB2α and GFP-ASB2αLA expression vectors for 20 hours. 20-μg aliquots of the protein extracts were separated by SDS-PAGE and immunoblotted for GFP, FLNa, JAK1, JAK2 and NM myosin IIA (A). Cells were fixed and stained for JAK2 and FLNa (B). Scale bar represents 10 μm. (C) Ba/F3 cells were nucleofected with the GFP-ASB2α expression vector for 20 hours. After fixation and permeabilization, cells were stained with anti-JAK2 or anti-FLNa and brilliant violet 421-conjugated anti-rabbit antibodies. FACS analysis of GFP positive cells (bold black lines) and GFP negative cells (light grey areas) is shown. Dark grey areas represent staining profiles with secondary antibodies alone.

We next examined degradation of JAK proteins in lymphoid lineage cells. GFP-ASB2α or GFP-ASB2αLA was transiently expressed in murine Ba/F3 pro-B lymphocytes (Figure 2). At 20 hours after transfection, western blotting showed that the level of FLNa was reduced in the presence of ASB2α but not in the presence of the E3 ubiquitin ligase defective mutant ASB2αLA (Figure 2A). In contrast, levels of JAK1, and JAK2 were similar in cells expressing wild-type ASB2α or the inactive ASB2αLA mutant. To confirm these results, the expression of JAK2 and FLNa was analyzed by immunofluorescence microscopy in GFP-ASB2α- and GFP-ASB2αLA-expressing cells. Indeed, ASB2α did not induce degradation of JAK2 while inducing degradation of FLNa (Figure 2B). To additionally confirm these data, the expression of JAK2 or FLNa was determined together with the respective GFP-ASB2α expression by FACS analysis. Figure 2C shows the expression profiles of JAK2 and FLNa in GFP negative and GFP positive Ba/F3 cells transfected with GFP-ASB2α expression vector. Although FLNa expression was dramatically reduced in GFP-ASB2α-expressing cells, levels of JAK2 were similar in GFP negative and GFP positive cells. Collectively, our results indicate that ASB2α E3 ubiquitin ligase does not trigger degradation of endogenous JAK1 and JAK2 proteins in Ba/F3 pro-B lymphocytes.

**Figure 3:**
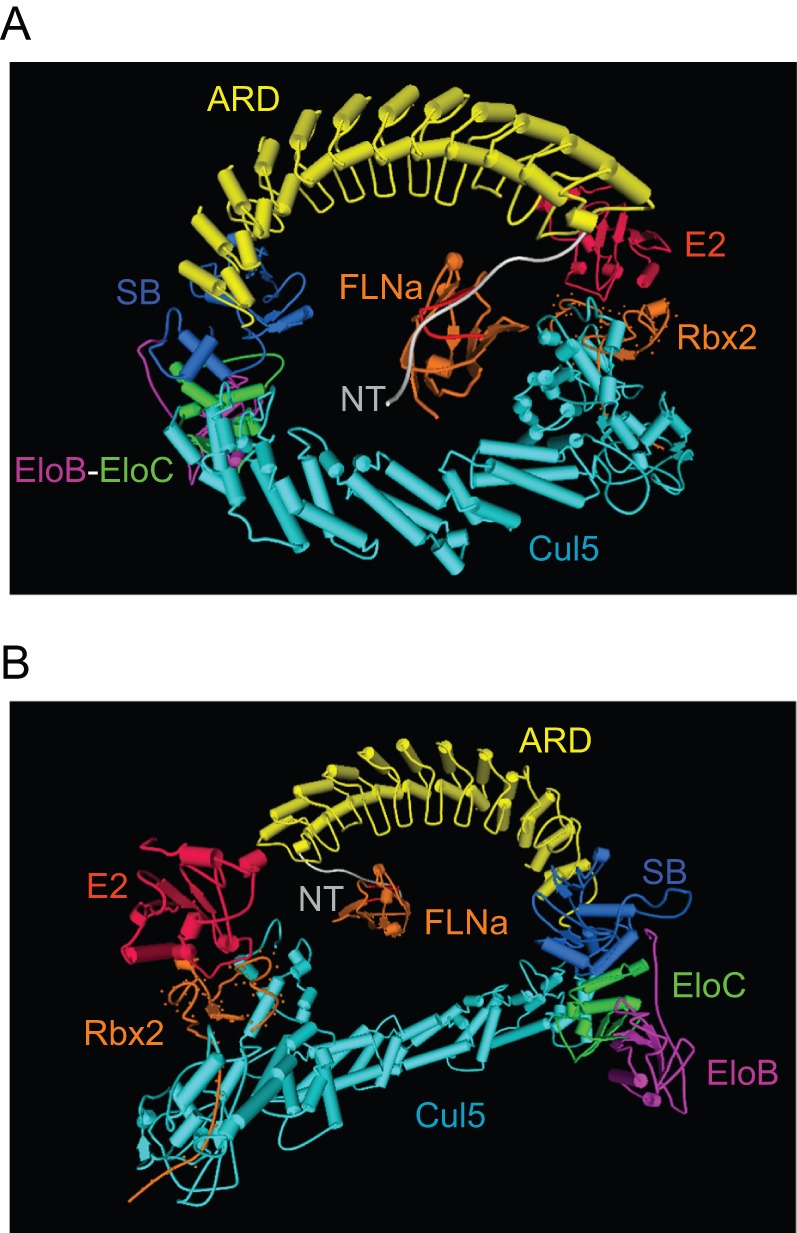
Modeled structure of the ASB2α E3 ubiquitin ligase complex. (A) The ASB2α N-ter peptide (NT) linked to the 12-ankyrin repeat domain (ARD, yellow color-coded) is docked into the immunoglobulin-like domain 21 of FLNa (FLNa). The ASB2α C-ter SOCS box (SB, blue color-coded) is docked into EloC. The adaptor Cul5 forms a bridge between EloC and Rbx2 RING domain, resulting in the E3 ligase complex. Rbx2 binds an E2 ubiquitin conjugating enzyme, the Connoly surface of interaction being shown as small dots. (B) Display of the same macromolecular complex as in panel A, after 180° rotation around the Y axis, allowing a better view of EloB and of the E2-Rbx2 interface.

It has been recently proposed that ASB2α but also ASB1, through its association with Skp2, bridges the EloB/EloC/Cul5/Rbx2 complex to the Skp1/Cul1/Rbx1 complex leading to the formation of non-canonical E3 ligase complexes involved in the polyubiquitylation and subsequent degradation of JAK2 [15]. Because this was observed following overexpression of ASB2α in non-hematopoietic cells and because the ASB2α/EloB/EloC/Cul5/Rbx2 complex drives *in vitro* polyubiquitylation of FLNa and FLNb [3], [6], ASB2α-induced polyubiquitylation of FLNs is unlikely to involve the Skp2 complex in physiological relevant settings. We performed molecular modeling to generate the first structural model of an E3 ubiquitin ligase complex of an ASB protein bound to its substrate. Indeed, ASB2α belongs to the family of ASB proteins that harbour a variable number of ankyrin repeats and a SOCS box located at the carboxy-terminal end of the protein. The high-resolution structure of the ankyrin R D34 region served to model the ASB2α 12-ankyrin repeat domain (ARD). A distinctive feature was a long insertion between repeats 10 and 11 which was submitted to a special loop optimization protocol. The ASB2α 44-residue SOCS box was modeled after that of SOCS2, the BC and Cul5 boxes forming a well-conserved helix-loop-helix structure. Since no significant homology could be detected with the intervening sequence between ASB2α ARD and SOCS box, this linker region was threaded to part of the *S. cerevisiae* ubiquitin conjugating enzyme 1, used as a topological template. The whole ASB2α was assembled, and its amino-terminal FLNa-binding motif was docked into the immunoglobulin-like domain 21 of FLNa as described for the free peptide [13]. The SOCS box was then docked into the EloC-EloB complex by homology to the crystallographic structure of the ternary SOCS2-EloC-EloB complex. The resulting interface of interaction with EloC predicts a critical role for Leu 548 from ASB2α BC box, in good agreement with the deficiency of the aforementioned ASB2αLA mutant regarding FLNa degradation. Finally, taking advantage of the homology between Skp1 and EloC, we used the crystallographic structure of the Cul1-Rbx1-Skp1-Skp2 SCF E3 ubiquitin ligase complex, not only as a source of atomic coordinates to model Cul5 and Rbx2 structures, but also as a structural framework to piece together the various components of the ASB2α E3 ubiquitin ligase complex bound to the immunoglobulin-like domain 21 of its substrate FLNa (Figure 3).

In conclusion, results from our group and from others indicated that, in hematopoietic cells, ASB2α did induce degradation of FLNs and MLL whereas it did not induce degradation of JAK proteins. Furthermore, one important objective and a major challenge for therapeutic means will be to predict the structural dynamics of functional E3 ubiquitin ligases. In this regards, our findings should have broad implications for our understanding of ASB2α mechanisms of action in hematopoiesis.

## Materials and Methods

### Cell lines, culture conditions

NB4 cells and PLB985 cells (a subclone of HL-60 cells) were used and differentiated with 10^−6^ M *all-trans* retinoic acid (Sigma-Aldrich) as described [6]. Clonal PLB985 cells stably transfected with ZnSO_4_-inducible vectors expressing wild-type ASB2α or an E3 ligase defective mutant of ASB2α (PLB985/MT-Flag-ASB2α and PLB985/MT-Flag-ASB2αLA cells, respectively) were cultured as described [6]. U937 were cultured in RPMI 1640 medium with 10% fetal bovine serum and 2 mM glutamine. Ba/F3 cells were cultured in the same medium supplemented with 10 ng/ml recombinant mouse interleukin-3 (Immunotools). Ba/F3 and U937 cells were transfected using the nucleofector V solution and the X001 and V001 programs, respectively, as recommended by the manufacturer (Lonza). Cells were then cultured for 20 hours.

### Cell extracts, antibodies, immunofluorescence microscopy

Whole cell extracts were prepared as described [6]. The rabbit serum against human ASB2α (1PNA) has been previously described [5]. Anti-FLNa (clone PM6/317) and anti-non-muscle myosin II heavy chain A were from Millipore and Covance, respectively. The anti-human FLNa antiserum that cross-reacts with mouse FLNa and used for immunofluorescence experiments has been previously described [6]. A novel rabbit anti-FLNa antiserum, raised against bacterially expressed recombinant human FLNa d16–20, and absorbed against the homologous fragments of human FLNb and FLNc, was used for flow cytometry. Anti-JAK1, anti-JAK2 and anti-JAK3 were from Cell Signaling Technology. Immunofluorescence analyses were performed as described [13].

### Quantification of JAK2 and FLNa expression by FACS

Cells were fixed in 3% paraformaldehyde in PBS and permeabilized 15 min in PMZ-T (1X PBS, 0.2% BSA, 0.3% Triton X100, and 50 mM NH_4_Cl). Cells were incubated at room temperature for 1 hour without or with antibodies against FLNa or JAK2, and then for 1 hour with brilliant violet 421-conjugated donkey anti-rabbit antibodies (Biolegend). Samples were analysed by flow cytometry using a LSRII cytometer (Becton-Dickinson).

### Molecular modeling

Modeling was performed using the *Accelrys* modules Homology, Discover, Docking and Biopolymer, run within InsightII (2005 version) on a *Silicon Graphics* Fuel workstation.

### Homology modeling of the ASB2α domains and E3 ubiquitin ligase proteins

Structural templates were searched using PSI-BLAST against the Protein Data Bank (PDB). Best templates for the ASB2α Ankyrin Repeat Domain (residues 21–424) and SOCS box (residues 544–587) were found to be the human R ankyrin D34 region (PDB code: 1N11; 63% homology) [18] and the human SOCS2 SOCS box (PDB code: 2C9W_A; 41% homology) [19], respectively. Templates for Cul5 and Rbx2 proteins were Cul1 (PDB code: 1LDK_A-B; 33% homology) and Rbx1 (PDB code: 1LDK_C; 40% homology), respectively. In the absence of significant sequence homology, the SeqFold threading method was applied to the sequence of the linker region (residues 425–543) between the Ankyrin Repeat domain and the SOCS box of ASB2α. A thorough analysis of the predicted folds indicated that part of the *S. cerevisiae* ubiquitin conjugating enzyme 1 (PDB code: 1FZY_A) could be an appropriate topological template. Sequence alignment between structural template and target protein was carried out using either the *Multiple_Sequence Alignment* or the *Align 123* program depending on the degree of homology, and served as an input for the automated homology modeling program *Modeler*. The modeled structures were submitted to a final Discover-driven energy refinement protocol, a prerequisite to subsequent docking. Models validity was assessed both by structural check and folding consistency verification using the respective *Prostat* and *Profiles_3D* programs.

### Modeling of an immunoglobulin-like domain 21 of FLNa bound ASB2α-E3 ubiquitin ligase complex

The whole ASB2α protein was assembled from its individual domains and N-terminal peptide within the Biopolymer module, and the FLNa-binding motif was docked into the immunoglobulin-like domain 21 of FLNa as described [13]. Modeling of the ASB2α-EloC-EloB complex was performed by homology to the high-resolution crystallographic structure of the SOCS2-EloC-EloB complex (PDB code: 2C9W) [19]. ASB2α SOCS box was superimposed onto the SOCS2 SOCS box (2C9W_A), and replaced it. Keeping EloC (2C9W_C) and EloB (2C9W_B) in place, ASB2α SOCS box was docked into EloC, following the aforementioned *Affinity*-driven protocol. Modeling of the ASB2α-EloC-EloB-Cul5-Rbx2 complex was conducted by homology to the crystallographic structure of the Cul1-Rbx1-Skp1-Fbox^Skp2^ SCF ligase complex (PDB code: 1LDK) [20] which was used as a structural framework. In a first step, the respective homology-derived structures of Cul5 and Rbx2 (see above) were superimposed onto Cul1 (1LDK_A-B) and Rbx1 (1LDK_C), replacing them in the complex. Rbx2 was docked into Cul5. In a second step, EloC in the ASB2α-EloC-EloB complex was superimposed onto Skp1 (1LDK_D) from the initial SCF ligase complex, and replaced it. EloC was docked into Cul5. Finally, the UbcH7 E2 was docked onto the Rbx2 RING domain based on the c-Cbl-UbcH7 structure (PDB code: 1FBV).
